# More than meets the eye: phenomenological insights into the functioning of people with lipoedema

**DOI:** 10.1080/17482631.2025.2463157

**Published:** 2025-02-20

**Authors:** Lise Maren Kloosterman, Renske Eilers, Aldo Scafoglieri, Ad Hendrickx, Rienk Dekker, Harriët Jager-Wittenaar

**Affiliations:** a Research Group Healthy Ageing, Allied Health Care and Nursing, Hanze University of Applied Sciences, Groningen, The Netherlands; b FAITH Research, Groningen, The Netherlands; c Center of Expertise for Lymphovascular Medicine, Nij Smellinghe Hospital, Drachten, The Netherlands; d Research Group Experimental Anatomy, Department of Physiotherapy, Human Physiology and Anatomy, Faculty of Physical Education and Physiotherapy, Vrije Universiteit Brussel, Brussels, Belgium; e Research Group Nursing Diagnostics, Hanze University of Applied Sciences, Groningen, The Netherlands; f Department of Rehabilitation Medicine, University of Groningen, University Medical Center Groningen, Groningen, The Netherlands; g Department of Gastroenterology and Hepatology, Dietetics, Radboud university medical center, Nijmegen, The Netherlands

**Keywords:** Lipoedema, functioning, phenomenological qualitative study, ICF, Interpretative phenomenological approach

## Abstract

**Purpose:**

The aim of this study was to explore the functioning of people with lipoedema from their perspective.

**Methods:**

This was a qualitative study following a phenomenological perspective using individual in-depth interviews with a convenience sample of 13 Dutch people with lipoedema. The data were analysed by using an interpretative phenomenological approach.

**Results:**

Two overarching group experiential themes (GETs) were identified: (1) “physical complaints are only a part of their problems”, and (2) “longing for improved appearance and functioning”. The GETs were composed of the following subordinate GETs: “disproportionate functioning of the lower body”, “navigating daily life with lipoedema”, “coping with uncertainty”, “the toll of shame and stigmatization”, “consequences of a very negative self-image”, “desire for change and improved appearance and functioning”, and “an ongoing journey of acceptance”.

**Conclusions:**

Participants experience that physical complaints affect daily activities, but with adequate adaptability, participation issues are relatively limited. Instead, shame about their appearance and (fear of) stigmatization mainly leads to social avoidance. Additionally, participants long for freedom to do what they want, wear what they want, and desire a different appearance. Therefore, the experienced level of acceptance of the condition is context- and situation-dependent and not straightforward, which is complicated by the burden of knowing that the condition is chronic.

## 1. Introduction

Lipoedema is a chronic condition associated with a bilateral disproportionate increase in adipose tissue in the legs, thighs, buttocks, and sometimes in the arms (van Esch-Smeenge et al., 2017). People with lipoedema often experience symptoms such as a sensation of heaviness, (pressure) pain, and tenderness (Faerber et al., 2024). Lipoedema was first reported in 1940 by Allen and Hines (Wold et al., 1951), but the etiopathogenesis of lipoedema remains unclear. This condition particularly affects women, with problems often arising at puberty, pregnancy, or during menopause (Bertsch et al., 2020; Child et al., 2010; Paolacci et al., 2019). Lipoedema in men is very rare and has only been described in a few cases. Male individuals with lipoedema usually have an underlying condition associated with higher oestrogen levels or lower testosterone levels (Buso et al., 2019). Lipoedema is diagnosed on the basis of clinical evidence and the exclusion of differential diagnoses (Halk & Damstra, 2017). Due to the lack of consistent diagnostic criteria and its frequent co-occurrence with obesity, lipoedema is often misdiagnosed or confused with other conditions (e.g., lymphoedema and lipohypertrophy), leaving its prevalence unclear (Bertsch et al., 2020; Czerwińska et al., 2021).

People with lipoedema may encounter difficulties with daily functioning, which can be attributed to various factors such as pain (Dudek et al., 2016; Karayannis et al., 2017), reduced physical capacity (Halk & Damstra, 2017; van Esch-Smeenge et al., 2017), as well as the existence of psychological issues like depressive and eating disorders (Erbacher & Bertsch, 2020). To understand an individual’s functioning, the World Health Organization (WHO) has developed the International Classification of Functioning, Disability, and Health (ICF) (World Health Organization, 2001). WHO defines functioning as “an umbrella term for body functions, body structures, and activities and participation”, and describes functioning as “the result of interactions between a person’s health status and environmental and personal factors” (World Health Organization, 2001). Our scoping review showed that current research on lipoedema mainly focuses on outcomes that fit within the ICF domains of body functions and body structures (Kloosterman et al., 2023). In the ICF domain activities, participation and environmental factors remained relatively underexposed. They were being studied in less than 18% of the included studies, while the domain of personal factors was studied in only half of the included studies.

Of the limited studies on the functioning of people with lipoedema, particularly regarding activities, participation, and environmental and personal factors, only four qualitative studies have explored this topic to date (Christoffersen & Tennfjord, 2023; Dahlberg et al., 2024; Fetzer, 2020; Melander et al., 2022). One study highlighted how lipoedema significantly restricted daily activities for diagnosed individuals, who felt controlled by their condition and lacked adequate professional support (Melander et al., 2022). The other studies highlighted healthcare providers’ misunderstanding and stigma surrounding lipoedema, emphasized the extensive impact of lipoedema on various aspects and quality of life, and noted the profound impact of lipoedema on future prospects (Christoffersen & Tennfjord, 2023; Dahlberg et al., 2024; Fetzer, 2020). However, in two of these studies, it is unclear whether the participants had the correct diagnosis (Christoffersen & Tennfjord, 2023; Fetzer, 2020).

No studies have provided a deep understanding of how people with lipoedema perceive their functioning across all domains of the ICF nor has sufficient research explored the meanings they attribute to their experiences. Gaining insight into their functioning will provide a valuable foundation for optimizing treatment and coping strategies. Therefore, the aim of this qualitative study was to explore the functioning of people with lipoedema from their perspective.

## 2. Methods

A qualitative study with a phenomenological perspective was conducted to understand functioning in people with lipoedema from their point of view. The functioning of people with lipoedema was generally defined according to the ICF framework in terms of body functions and body structures, activities and participation, environmental factors, and personal factors (World Health Organization, 2001). Using a qualitative approach allowed us to explore the perspectives of people with lipoedema regarding their functioning, with attention to their lived experiences on emotional, physical, and contextual levels. By systematically asking participants about these topics, we gained a deeper understanding of their lived experiences. This approach provided insights into the “what”, “how” and “why” of functioning as perceived by people with lipoedema, as this has not yet been investigated (Moustakas, 1994). We reported on this study according to the items of the Consolidated Criteria for Reporting Qualitative Research (Tong et al., 2007).

### 2.1. Participants

A convenience sample of Dutch people with lipoedema was asked to participate in this study to gather information-rich cases (Creswell & Poth, 2017). The study included individuals willing to share their experiences and diagnosed with lipoedema by an experienced dermatologist at the Expertise Centre for Lymphovascular Medicine (ECL) at Nij Smellinghe Hospital in Drachten, The Netherlands. This particular inclusion criterion was chosen because lipoedema is frequently misdiagnosed or mistaken for other conditions, mainly due to the absence of standardized diagnostic criteria (Bertsch et al., 2020). The diagnosis was based on the following criteria: bilateral and symmetrical disproportionate fat distribution; persistent disproportionate fat distribution despite weight loss or elevation of extremities; pain, tenderness, and easy bruising; and minimal pitting oedema (Wold et al., 1951). Individuals who did not have proficiency in Dutch or English and individuals who faced challenges in communicating, reading, and writing were excluded from participating in the study.

### 2.2. Recruitment

The participants were recruited at the ECL. A physiotherapist informed the participants about the study during their visit to the ECL and provided them with a participant information form. The physiotherapist also gained permission from the participants to allow the researcher to contact them. Interested participants were then assessed for eligibility based on both inclusion and exclusion criteria, as mentioned above. Those who met the criteria and expressed their willingness to participate were invited to participate in the study. The participants were given the choice to opt for either an in-person interview or an online interview. Before conducting the interviews, each respondent provided informed consent. An envelope holding two copies of the participant informed consent forms and a questionnaire (Appendix I) was sent to the participants. They were requested to complete and return one signed copy of the informed consent form along with the questionnaire before the interview. This questionnaire encompassed questions regarding participants’ demographics. All participants consented to participate in the study and be audio-recorded during the interview.

### 2.3. Data collection

Eleven online and two face-to-face in-depth interviews were conducted in Dutch between February 2023 and February 2024. In-depth interviews were chosen as a suitable method because they enabled the researcher to explore various aspects of the participants’ lives, allowing them to share personal experiences and providing insight into their understanding of and experiences with daily functioning (Liamputtong & Douglas, 2005; Steinar, 2007). Semi-structured interviews were used to yield rich data, and an interview guide was used to coordinate the interview (Appendix II). Questions were formulated regarding each domain of the ICF to invite participants to share their experiences and feelings about those domains. For example, “Can you tell us if you feel that having lipoedema affects your activities? When you think about your surroundings, such as other people or your living environment, are there aspects that help you with your functioning?”. The questions were formulated in an open-ended manner to provide the respondent with the maximum opportunity to answer in any direction. In-depth follow-up questions were used to gain more profound insight into the previously given perceptions. Before conducting the first interview, the guide was tested with an individual with lymphoedema who received inpatient treatment at the ECL. During the pilot interview, no individuals with lipoedema were available, so a similarly aged woman with lymphoedema participated, as both conditions share comparable challenges in daily life. This resulted in adjustments to enhance the quality of the questions in the interview guide, including their wording, structure, and content. The study incorporated a combination of face-to-face interviews and online interviews to achieve a broader geographical representation of participants, ensuring a diverse range of perspectives (de Villiers et al., 2021). The average duration of the interview was approximately 1 hour. The interviews were conducted by a single researcher (LMK), an educated physical therapist, and a clinical health scientist trained in conducting interviews. During the interviews, the researcher took field notes to record descriptions of the observed non-verbal communication (e.g., facial expressions, pauses), reactions (e.g., sighs, voice changes, sadness), and her own feelings. Data collection continued until saturation was reached, indicating that an interview no longer yielded experiential statements that had not been mentioned before.

### 2.4. Data analysis

The interviews were transcribed verbatim. The data were analysed by using ATLAS.ti 23 (Scientific Software Development GmbH, 2019) using an interpretative phenomenological approach (IPA) according to the steps of Smith et al (Smith et al., 2022). In the analysis process, seven distinct steps were identified: (1) reading and rereading the transcript, (2) exploratory noting, (3) constructing experiential statements, (4) searching for connections across experiential statements, (5) naming the personal experiential themes (PETs) and consolidating and organizing them, (6) continuing the individual analysis of other cases, and (7) working with PETs to develop group experiential themes (GETs) across cases. The authors chose to include quotes in the exploratory noting phase only when it was beyond reasonable doubt that participants were reflecting on their functioning with lipoedema, either by directly reacting to a question regarding their lipoedema or when they were directly talking about their lipoedema themselves.

The first three steps of the first four interviews were independently analysed by two researchers (LMK and BvdA). In a consensus meeting, both researchers reached an agreement on the exploratory notes and experiential statements within the transcripts. However, due to personal circumstances, the independent researcher (BvdA) had to withdraw from the task. As a result, LMK undertook the first three steps of the analysis for the remaining transcripts by herself. To ensure that the researcher made accurate interpretations of the interviews, all participants were asked to verify the experiential statements through member checks. In subsequent meetings with a senior researcher and expert on qualitative research (RE), the personal experiential themes, and GETs were further refined, and any disagreements or ambiguous quotations were thoroughly discussed among all authors. The Dutch quotes and statements used in this article were translated into English by someone with a bilingual upbringing and strong English writing skills.

### 2.5. Trustworthiness

Several measures were taken to enhance the credibility and confirmability of this study (Elo et al., 2014). Throughout the research process, reflexivity played an important role. Bracketing was used during data collection and analysis to minimize subjectivity by identifying and acknowledging the researcher’s personal beliefs and assumptions (Chan et al., 2015). Additionally, 30% of the first three steps of the data analysis were independently conducted by two researchers and subsequently peer-reviewed by members of the research team. The subsequent steps within the data analysis were all peer-reviewed by a senior researcher and expert in qualitative research, along with all other authors. Furthermore, the interviews were transcribed verbatim to reduce the risk of bias. Memo taking was also employed throughout all stages of the study, to record the researcher’s reflections and detail the context and important aspects of the interviews. To enhance transferability, a comprehensive description of participants’ characteristics and outcomes was included (Elo et al., 2014). To enhance credibility, member checks were employed by sharing the participants’ specific experiential themes with all the participants (Boeije, 2010). Furthermore, the researcher tried to establish a strong rapport with the participants during the interviews by explicitly stating that there were no correct answers to the questions and conducting the interviews at participants’ preferred locations (Horsfall et al., 2021). Additionally, a transparent analytical process was followed, adhering to specified steps by Smith et al. (2022).

### 2.6. Ethical considerations

Recruitment and data collection started subject to the approval of the study by the Hanze Ethics Advisory Committee (approval number: heac.2023.003) and Nij Smellinghe Hospital’s local feasibility committee (reference: BK/HdJ/ID 23,561). All women who participated in the study were provided with information about the study’s purpose and objectives, their right to privacy, the secure storage of collected data, and the option to withdraw from the study at any time. To protect participants’ privacy, personal data were de-identified, processed confidentially, and secured using established and accepted data security protocols. Written records were managed in a comparable manner and securely stored. The signed informed consent forms and interview transcripts were stored in compliance with the regulations outlined in the General Data Protection Regulations. Furthermore, the study was conducted following the principles stated in the Declaration of Helsinki.

## 3. Results

We interviewed 13 individuals medically diagnosed with lipoedema. The characteristics of these participants are displayed in Table I. All participants were women, ranging in age from 24 to 63 years. The onset of lipoedema symptoms occurred predominantly during adolescence. Most women experienced lipoedema affecting areas from the buttocks to the ankles, often in combination with the arms, and had one or more comorbidities.

**Table I.** Participant characteristics.

Age in years, mean, minimum - maximum	42, 24–63
Female gender, percentage of participants (n)	100% (13)
Onset of lipoedema, percentage of participants (n)	
Before the age of 10 years	7.7% (1)
Adolescence (10–19 years)	84.6% (11)
Older than 19 years	7.7% (1)
Anatomical location lipoedema fat, percentage of participants (n)	
Lower legs	15.4% (2)
Upper legs	15.4% (2)
Lower and upper legs	69.2% (9)
Involvement of the arms	53.8% (7)
Positive family history, percentage of participants (n)	30.8% (4)
Diagnosed with at least one comorbidity*, percentage of participants (n)	53.8% (7)

*reported comorbidities: attention-deficit disorder, attention-deficit/hyperactivity disorder, asthma, irritable bowel syndrome, migraine, osteoarthritis, pervasive developmental disorder not otherwise specified, postural orthostatic tachycardia syndrome, shingles, varicose veins.

The experiences of the participants revealed two GETs (Table II). The first GET is “physical complaints are only a part of their problems”, with subordinate GETs: disproportionate functioning of the lower body; navigating daily life with lipoedema; coping with uncertainty; the toll of shame and stigmatization; and consequences of a very negative self-image. The second GET is “longing for improved appearance and functioning”, with subordinate GETs: desire for change and improved appearance and functioning, and an ongoing journey of acceptance.

**Table II.** Group experiential themes.

Group experiential theme:	Physical complaints are only a part of their problems
Subordinate group experiential themes:	Disproportionate functioning of the lower body
	Navigating daily life with lipoedema
	Coping with uncertainty
	The toll of shame and stigmatization
	Consequences of a very negative self-image
Group experiential theme:	Longing for improved appearance and functioning
Subordinate group experiential themes:	Desire for change and improved appearance and functioning
	An ongoing journey of acceptance

### 3.1. Physical complaints are only a part of their problems

Functioning with lipoedema involves more than just dealing with physical complaints. Participants experience several physical complaints due to lipoedema, but significant shame and perceived stigma also have substantial consequences within their social environment. Additionally, uncertainty about future functioning plays a role. The extent to which physical problems impact activities varies and is influenced by individuals’ ability to make adjustments, which is related to their personality and their ability to organize activities at their own pace.

#### 3.1.1. Disproportionate functioning of the lower body

Regarding their physical abilities, participants feel like their legs are not working properly and not keeping up with the desired manner and pace, making the lower body a burden. For instance, one participant indicated that during sports, she can strain her upper body more than her lower body.

Eh, I’m putting a strain on my upper body, for example, at four, and then my legs are just a bit lower, a bit less. Eh, they, um, yeah, they’re not really contributing as much or something. [.] Eventually, eh, yeah, it’s just not comfortable and then it chafes. (Participant 6)

She experiences discomfort from her skin and tissue getting in the way and causing chafing. Another participant mentions that she feels limited in her walking abilities, which poses a significant challenge in life as it prevents her from doing what she wants:

I can’t walk, I can’t move around like I want to. […] I just can’t do what I want to do. My legs don’t work very well, that’s just a really big problem for me. (Participant 7)

Participants explain that they feel pain and fatigue in their legs, which is annoying and unpleasant and, moreover, makes everyday activities and movements difficult. Additionally, one participant described how the constant presence of pain causes sadness. She explains that no matter what she does, the pain is always there. Furthermore, participants feel that they cannot function “normally” and below what would be expected from someone in good health.

Then I already felt, my legs feel different than my arms. [.] I’ve always felt that this is not, it’s not, eh, it doesn’t function normally, not like you would expect from a healthy person, so to speak. (Participant 12)

#### 3.1.2. Navigating daily life with lipoedema

The described pain and discomfort leads to making concessions in daily life activities and having to adapt accordingly. Some participants find it easier to adjust than others. For example, one participant says she stopped playing sports because it was no longer enjoyable when she could no longer participate fully.

You know, when I work out and I can’t do one or two things, that’s not a big deal. But when you go to work out, and you can’t really participate half of the time, then it’s just not fun anymore. (Participant 7)

Another participant is able to make adjustments and can participate in an activity by biking instead of walking to still engage in family activities. For others, their physical discomfort serves as an extra motivation to become more active.

Participants indicate that they made adjustments. Adjustments to activities are sometimes made consciously by participants but often unconsciously. For instance, one participant mentioned that she probably just consciously or unconsciously chose activities that suited her. Another notes that, having lived with lipoedema for so long, she likely does everything differently. Dealing with lipoedema for several years makes participants feel more accustomed to making adjustments. For some participants, the ability to adjust seems effortless, resulting in lesser participation problems, while for others, it is a quest that elicits ambivalent feelings. One woman explained it as follows:

So you’re constantly dealing with little things, and maybe I’ve always done that, to change my life a little bit each time. Adjusting my life to it. And that’s not bad because they’re all small things, I think. But in the end, it all adds up. [.] Yeah, I might be adjusting myself too much, but then I quickly think, yeah, that’s just how it is. But I’m actually kind of bummed about it too. (Participant 1)

While some participants find it easier to adapt because it allows them to do activities they are passionate about, others feel frustrated because they must adapt activities to what they can do rather than what they would like to do. Besides being able to adapt effectively, participants indicate that their character and perseverance help them continue or attempt activities as much as possible. They explain that finding the right balance between physical, mental, and emotional workload and the body’s ability to handle this workload is crucial for engaging in activities. They do this by balancing rest and exertion at home and work, setting boundaries, and breaking tasks into smaller parts. For example, one participant explained that she skips soccer practice to ensure that she has more energy for other activities during the week. Other participants pace activities at work to endure an entire workday better or allow themselves more time and energy to complete household tasks.

#### 3.1.3. Coping with uncertainty

Participants express uncertainty and fear for the future, because they perceive a progressive decline in their abilities and the associated physical discomfort. This manifests in various ways. For example, participants are afraid of not being able to walk anymore:

I find that really hard. That’s also my biggest fear actually, that I, because I can already walk less. (Participant 7)

However, for some, this fear also serves as motivation to improve functioning. For instance, one participant mentioned feeling that her physical complaints are increasing and that she needs to improve her physical abilities to prevent her condition from worsening. Participants are afraid that their abilities will be increasingly limited as they age with lipoedema. One participant expresses uncertainty about the effects of menopause and the associated hormonal fluctuations on her lipoedema. Despite her anxiety, she also expresses pride in how much she exercises:

I am proud of the extent to which I stay active and how I do it, and I also believe that if I hadn’t done all of that, my lipoedema would have been much worse. [.] My hope is that I can keep it under control like this and that it stays this way, but I’m a bit concerned. The menopause is coming, and I think, “Yeah, that’s of course another hormonal fluctuation, what will that do?” (Participant 4)

At the same time, she worries about whether she will be able to maintain her level of physical activity as she ages, as it allows her to function at her current level.

#### 3.1.4. The toll of shame and stigmatization

Participants express feeling shame about their appearance because of the lipoedema. Because of this, they avoid social activities and withdraw from social interactions because they feel vulnerable and uncomfortable. For instance, participants avoid the swimming pool or visiting the beach out of embarrassment about their appearance and fear of comments from others. One participant expressed deep sadness about avoiding activities she once enjoyed due to her appearance:

I used to love going to the beach. I really enjoyed swimming in the sea too. But, eh, no, we’re not doing that anymore. Well, that’s obviously really sad. Yes, I think that’s really awful. (Participant 5)

Another participant indicates that she feels ashamed of her stockings, which caused her to withdraw from social interactions and activities. This manifests in not going to play hockey despite she wants to and avoiding conference visits with her colleagues in the summer because her stockings are visible in both activities.

Beyond internal shame, participants also feel stigmatized and fear to be stigmatized due to their appearance. Like feeling ashamed, this also leads to avoidance behaviour and imposes limitations in the social domain and in daily intimate interactions with a partner, as they anticipate others’ thoughts about them:

When I’m in the shower, he really shouldn’t open the door for me. It makes me so angry. He says: ‘Come on, stop it.’ But I just feel so uncomfortable with that. So, eh, when I go to bed, I turn off the light before I undress. Of course, he can feel it, how it is, [.] but when he puts his leg over mine, I just think: yeah, that’s right, all those bumps. You’re constantly aware of it. (Participant 11)

One participant expresses feeling more comfortable in an environment with more overweight people, which offers comfort and enables participation in social activities. She notes that in her family, where more people are overweight, nothing is organized that she cannot participate in. Additionally, resilience, strong self-confidence, and the ability to put things into perspective help participants counteract negative feelings of shame and stigmatization. However, this does not always lead to performing the activity anyway. For instance, one participant avoids the sauna or swimming pool because of her appearance but feels strongly that people must accept her as she is. Another participant feels socially excluded at the gym but remains confident about her appearance and is unaffected by others’ remarks. Despite the negative influence of shame and stigma, especially in social activities, social contacts are perceived as helpful for functioning. Recognition, understanding, and encouragement from the social network promote functioning and well-being, as well as the acceptance of limitations and necessary adjustments.

#### 3.1.5. Consequences of a very negative self-image

Shame about appearance leads to a constant awareness of one’s legs, a negative self-image, and low self-confidence. One participant indicates that she is very aware of the physical space she occupies and often notices people looking at her legs, which makes her feel self-conscious and uncomfortable:

Well, when I’m on the bike, you know, I see people just looking at my legs. That’s something I notice, and I don’t believe that they think, ‘Oh, what a nice pair of glasses that lady has’ or something. No, I really see them looking at those legs. […] I’m aware of the space I take up. (Participant 5)

Additionally, a negative self-image results in avoiding mirrors. One participant states that she is disgusted by her appearance, cannot be who she wants to be, and that this greatly impacts her self-confidence.

And then I come to the gym, and there are mirrors everywhere, from top to bottom [.] And in that moment, I feel disgusted by myself. So incredibly ugly. Gross. I really do say that to myself. It’s awful. I absolutely hate my legs. They’re just unbearable to look at. (Participant 11)

Mirrors in her home are positioned so that only her face is visible. Concealing one’s appearance with appropriate clothing is also common among these participants. For example, one participant mentions that although she does not find herself attractive, she tries to look well-groomed and neat with the help of makeup and clothing. Coping with a constantly changing and less attractive appearance is perceived as challenging for these participants and leads to anxiety about self-image and future body size.

### 3.2. Longing for improved appearance and functioning

Participants explain that they have various desires related to their functioning, with the desire for a different appearance and improved functioning being the most prominent. The acceptance of having lipoedema is therefore challenging, and the degree of acceptance varies over time. Even if people experience acceptance to varying degrees, it does not mean they do not wish things were different. Additionally, the chronic nature of the condition plays a role in this and takes its toll.

#### 3.2.1. Desire for change and improved appearance and functioning

Participants describe a longing for changes in their functioning and appearance due to the perceived physical and emotional limitations imposed by lipoedema. One participant expresses desires for freedom, such as wearing preferred clothing, not constantly monitoring her diet, and participating in activities without limitations.

I’d also like to be able to wear a skirt when the weather is nice, or, you know, when it’s 40 degrees, not have to think, ‘just let me work because there’s air conditioning,’ but instead feel free to wear what I want without constantly worrying about what I should put on. (Participant 4)

Another participant regrets that her lipoedema prevents her from enjoying activities she otherwise could have. The symptoms and limitations restrict her freedom. Despite growing older and finding some level of acceptance, the desire for a different appearance persists for some participants:

I’m generally happy with myself; it’s not that I’m unhappy. But, you know, there are definitely things I’d like to change. [.] If they told me today, ‘You can have it done tomorrow, and it’s covered,’ I’d have it done one after the other. (Participant 1)

Others feel sadness from not functioning normally and distress when their legs do not work as they wish, preventing them from doing what they love or moving as they would like. One participant mentions that her size has always been a hindrance and that she always feels different from the norm.

Mentally, my size has always been a barrier. You’re always perceived as different. (Participant 5)

Another participant wishes she could disregard the unpleasant stigmas she experiences at the gym when she visits the gym alone. In addition to the desire for change, participants also express a desire to prevent symptoms from worsening, and maintain their ability to do the things they find important in life. One participant experiences her lipoedema symptoms as relatively mild at present but worries that they might worsen, expressing relief that they are stable for now. Hopes are pinned on treatments like liposuction to improve both function and appearance, with some seeing liposuction as the only real solution.

#### 3.2.2. An ongoing journey of acceptance

Living with a chronic condition involves a constant quest for acceptance. One participant expresses an inner struggle over whether to undergo liposuction to alleviate symptoms and reduce stigma, even though she has come to love her body:

It’s taken me quite a while to embrace this non-standard body, and now I love it a lot. [.] The idea of no longer experiencing pain and, uh, fitting more easily into clothes, and dealing less with the stigma is, yeah, very appealing. (Participant 8)

Another participant feels that accepting lipoedema is easier when understanding that it is a condition that develops in the body, and that she does not need to blame herself. However, acceptance remains a continuous effort, especially when compounded by remarks from others about her appearance. Furthermore, acceptance is not always straightforward; it depends on certain conditions. For instance, one participant is content with her current level of functioning and can accept it, but only if it remains stable. Another participant can accept her limitations but struggles with accepting her appearance. She mentions that she can manage her limitations by incorporating rest and organizing her day effectively, but she would immediately get new legs if that were possible. One participant explained it as follows:

I have accepted that I have it, but I do not accept that it is staying this way. […] Look, I know that my legs won’t look like an average person’s; I have accepted that, but I really want it to improve. (Participant 11)

The degree of acceptance also depends on the specific context and the physical and mental state at any given moment. A participant mentions that acceptance is easier when she feels better mentally, but when she is not feeling well, acceptance becomes more difficult. Additionally, knowing the condition is chronic takes a mental toll, as one participant stated:

Well, I find that quite intense. Um, but it’s also related to your overall body, meaning you always have to keep working on it to feel good. (Participant 5)

Participants feel that it is challenging to face the same problems every day and expressed disappointment when noticeable results are absent despite hard work on self-improvement. They feel that maintaining the necessary behavioural changes required for living with the condition poses a significant challenge. However, participants express that the ability to accept the condition and its limitations is aided by self-confidence, personality, and ageing, which helps mitigate the impact of lipoedema on daily functioning for some individuals.

## 4. Discussion

The aim of this qualitative study was to explore the perceived functioning of people with lipoedema from a phenomenological perspective. The findings from this research show that the people with lipoedema who were studied experience physical complaints as only part of the broader challenges they face in daily life. Lipoedema and its associated physical complaints affect daily activities, but with adequate adaption, it leads to relatively limited participation issues. Instead, shame about their appearance, the feeling of being stigmatized, and the fear being stigmatized mainly lead to avoidance behaviour in social activities and social avoidance. Additionally, women with lipoedema express a desire for things to be different in the form of a longing for a sense of freedom to do what they want to do and wear, the desire to just be normal, and the desire for a different appearance. The experienced level of acceptance of the condition is therefore context- and situation-dependent and not always straightforward, which is complicated by the burden of knowing that the condition is chronic.

Our findings within our population suggest that, for some women, challenges with functioning due to shame and perceived stigma in the social domain may outweigh the physical complaints in the physical domain. The avoidance behaviour due to the feeling of shame and stigmatization can be explained by the phenomenon of weight bias internalization (WBI) or self-stigma. This happens when someone is aware of having a stigmatized identity and internalizes the associated stereotypes (Corrigan et al., 2009). Research shows that greater WBI is associated with poorer physical, psychosocial (e.g., social functioning), and behaviour-related health (Romano et al., 2023). Furthermore, research suggests that people with high WBI often have a Caucasian background, a higher BMI, perceive themselves as heavier, have previous experiences with weight stigma, and are actively engaged in weight loss attempts, making WBI likely highly applicable to the lipoedema population studied in this research (Puhl et al., 2018). Additionally, a scoping review of people with chronic illnesses found that stigma is associated with social isolation, sadness, depression, anxiety, and reduced self-esteem, self-worth, and self-efficacy (Niño De Guzmán Quispe et al., 2021).

The findings of this study regarding stigmatization and shame align with previously conducted qualitative studies in this population. For instance, a study conducted in Sweden highlights experiences of fat-shaming and the feeling of being watched by others (Melander et al., 2022). Similarly, another study underscores the challenges within intimate relationships due to shame about one’s appearance (Christoffersen & Tennfjord, 2023). Avoidant behaviour resulting from body shame and fear of comments from others was also found in another qualitative study conducted in Sweden (Dahlberg et al., 2024). Additionally the study found that participants handle acceptance of problems related to lipoedema and their appearance differently, with the authors noting that dealing with and accepting the pain was more challenging. In contrast, our research indicates that shame about one’s appearance leads to greater challenges.

Our finding that degree of participation problems resulting from the physical complaints associated with lipoedema varied can be explained by their varying ability of the participants to adapt and their experienced acceptance. Research indicates that cognitive and behavioural flexibility correlates with problem-solving, improved adaptation to stress and life events, and enhanced resilience against physical illness (Fresco et al., 2006). Moreover, research suggests that accepting illness and flexibility in coping strategies decrease psychological distress and aid in adapting to the disease (Fresco et al., 2006; McCracken et al., 2005, 2007). While the level of illness acceptance among the studied population varies depending on context and situation, and is not always straightforward, studies suggest that individuals who can accept pain more effectively may demonstrate greater emotional resilience in managing their condition (Kratz et al., 2007). For example, individuals with chronic pain who persist in their activities despite experiencing pain tend to exhibit better physical, psychosocial, and emotional functioning, whereas those who attempt to control their pain or seek support often experience relatively poorer functioning (McCracken et al., 2007).

### 4.1. Strengths and limitations

This study has various strengths. First, it is the first qualitative study with a phenomenological perspective on the functioning of people with lipoedema using IPA to explore their lived experiences. It provided insights into the domains of activities, participation, and environmental and personal factors within the ICF framework. Second, the use of the ICF framework facilitated the development of the interview guide and ensured that all domains of functioning were considered. Third, only people diagnosed with lipoedema were included in this study to provide as true-to-life a picture of the functioning of people with lipoedema as possible and to overcome bias of self-diagnosing or misdiagnosis by other professionals in studies on lipoedema. Fourth, several measures were taken to enhance the credibility of this study-like member checks, and a transparent analytic process according to the IPA methodology.

This study also has limitations that need to be considered. First, half of the included participants had one or more comorbidities besides lipoedema. This made it difficult to sometimes distinguish functioning with lipoedema from functioning with the comorbidity. However, this is a representative sample of the patient population in daily practice, where people with lipoedema often have comorbidities like obesity, highlighting the difficulties in diagnosing people with lipoedema due to the absence of clear diagnostic criteria (Bertsch et al., 2020). Second, potential selection bias was introduced by selecting a convenience sample for this study. These participants had recently visited the hospital for their complaints and are more likely to have more problems with their functioning than those who have not recently visited a healthcare professional. However, by strictly including diagnosed individuals, we tried to mitigate the problem and bias of self-diagnosing or misdiagnosis by other professionals. Third, it was challenging for the researcher not to fall back into the role of physical therapist during the study and to avoid excessive clinical reasoning, which could lead to overly directed responses from participants. To overcome this, constant efforts were made to identify and acknowledge the researcher’s biases, personal beliefs, and assumptions through bracketing during data collection and analysis. Additionally, the data analysis was peer-reviewed by a senior researcher and expert in qualitative research, along with the other authors.

While being aware of the possible limitations of conducting online interviews, the researcher did not notice a lesser rapport during these sessions. In all the interviews, participants shared their perceptions freely, and emotions, such as those expressed through facial expressions, could be acknowledged. This allowed for more follow-up questions. Therefore, it did not limit the richness of the qualitative data obtained.

### 4.2. Implications

Based on this study, several recommendations can be made. First, it is important for healthcare professionals working with people with lipoedema to be aware that the problems and limitations experienced are context-dependent. Lipoedema significantly impacts overall functioning, with the extent of this impact depending on the context and the individual’s personality traits and character. Second, healthcare professionals should recognize that shame, stigmatization, and weight bias internalization significantly impact the functioning of this population. Therefore, these factors must be taken into account during the diagnostic and therapeutic processes. Psychosocial issues should be addressed throughout the diagnostic process, and treatment should focus on helping individuals to cope with feelings of shame and stigmatization, as well as empowering their self-confidence to mitigate the negative consequences of these experiences. In the absence of population-specific interventions, healthcare providers could incorporate cognitive-behavioural therapy or psychoeducation into their treatment plans (Tsang et al., 2016; Zamiri-Miandoab et al., 2021). Third, interventions based on illness acceptance, and cognitive and behavioural flexibility should be considered for individuals with chronic pain associated with lipoedema. These interventions, which aim to enhance physical, psychosocial, and emotional functioning, need to be tailored or developed specifically for this population. Future studies should focus on evaluating the effectiveness of these multidisciplinary interventions specifically within the lipoedema population.

## 5. Conclusion

Women with lipoedema who were studied experience physical complaints as only a part of the problem they face in daily life. While lipoedema and its associated physical complaints affect daily activities, with adequate adaption, this results in relatively limited participation issues. Instead, shame about their appearance, the feeling of being stigmatized, and the fear of stigmatization primarily lead to avoidance behaviour in social activities and social avoidance. Additionally, people with lipoedema express a desire for change, manifesting as a longing for the freedom to do what they want, to wear what they want, the desire to just be normal, and the desire for a different appearance. The experienced level of acceptance of the condition is context- and situation-dependent and not always straightforward, complicated by the burden of knowing that the condition is chronic. These psychosocial issues should be addressed throughout the diagnostic and therapeutic processes. Alongside interventions based on illness acceptance and cognitive and behavioural flexibility, treatment should aim to empower individuals to mitigate the negative consequences of stigma and shame. Future research should focus on evaluating the effectiveness of these interventions within this population.

## Supplementary Material

Supplementary files LM Kloosterman_resubmission.docx
