# Supplementary material for: More than meets the eye: phenomenological insights into the functioning of people with lipoedema
Source: Int J Qual Stud Health Well-being. 2025 Feb 20;20(1):2463157. doi: 10.1080/17482631.2025.2463157 (PMC11843652; doi:10.1080/17482631.2025.2463157)
Supplement: Supplementary files LM Kloosterman_resubmission.docx [file ZQHW_A_2463157_SM0421.docx]

**Appendix I Questionnaire for participants (Translated from Dutch)**

Participant number:

| Gender: |  |
| --- | --- |
| Age: |  |
| Lipoedema: | When did your lipedema symptoms first appear?  *Check what applies to you.*  During puberty  During of after pregnancy  During menopauze  Other, namely:  How old were you when you received the diagnosis of lipoedema from your doctor?  In which locations do you experience lipedema symptoms?  *Check what applies to you.*  Buttocks and hips  From buttocks to knees  From buttocks to ankles  Arms  Lower legs  Other, namely:  Does anyone else in your family have lipedema besides you?  *Check what applies to you.*  Yes, namely: Mother  Grandmother  Sister  Daughter  Aunt  Niece  Other, namely:  No |
| Conditions | Do you have any other diagnosed condition(s) besides lipedema?  Yes, namely:  No |
| Therapy | Were you treated for your lipedema symptoms before coming to the expertise center in Drachten?  *Check what applies to you.*  Yes  If yes, what treatment(s) have you received?  (For example, physiotherapy, lymphedema therapy, compression stockings, massage)    No  If yes, are you currently receiving these treatments?  Yes  If yes, how often do you receive these treatments?  *Check what applies to you.*  Daily  Weekly  Monthly  Other, namely:  No |

**Appendix II Semi-structured interview guide**

Can you tell us about what it was like for you when you received the diagnosis of lipoedema?

Can you describe what a typical day looks like for you?

What activities do you usually engage in during a day? (Examples: household chores, caregiving, transfers)

Are there aspects that assist you in performing these activities?

Are there aspects that hinder you in performing these activities?

Can you indicate whether your activities have changed since your lipoedema symptoms?

If yes, can you tell us how your activities have changed?

If yes, how do you experience these changes?

Can you tell us if you feel that having lipoedema affects your activities?

If yes, can you tell us how lipoedema affects your activities?

If yes, which activities are affected by lipoedema for you?

To what extent do you feel that you can participate in all the activities you want to do?

Can you specify which activities you engage in outside the home? This can include sports, paid work, volunteer work, caregiving, or hobbies.

Are there aspects that help you in participating in these activities outside the home?

Are there aspects that hinder you in participating in these activities outside the home?

Can you indicate if participation in activities outside the home has changed since your lipoedema symptoms?

If yes, can you tell us how your activities outside the home have changed?

If yes, how do you feel about these changes?

Can you tell us if you feel that having lipoedema affects your activities outside the home?

If yes, can you tell us how lipoedema affects your activities outside the home?

If yes, which activities are affected by lipoedema for you?

To what extent do you feel that you can participate in activities outside the home?

Now, I would like to delve into physical functioning. By this, I mean the functioning of your body. This can include your general fitness and strength, as well as aspects like bowel movements and the functioning of your heart and lungs.

Can you tell us what you believe a healthy body should meet?

To what extent is this the case for you?

Are there things in your body that should work differently than they currently do?

Can you indicate if your physical functioning has changed since you received the diagnosis of lipoedema?

I would like to learn more about your mood and thinking. Can you describe how your mood generally is?

Can you indicate if your mood or thinking has changed since you received the diagnosis of lipoedema?

If yes, what has changed?

If yes, how do you experience these changes?

When you think about the functioning of your body or your mood and thinking, can you tell us if other people, such as family members, friends, colleagues, or neighbours, influence it?

If yes, can you tell us how these people influence these activities?

If yes, can you tell us who has an impact on your physical functioning or your mood and thinking?

Do other people, such as family members, friends, colleagues, or neighbours, influence the activities you do indoors or outdoors?

If yes, can you tell us how these people influence these activities?

If yes, can you tell us who has an impact on your indoor or outdoor activities?

Does having lipoedema affect your relationships with family members, friends, colleagues, or neighbours?

If yes, can you describe how having lipoedema affects your relationships?

When you think about your surroundings, such as other people or your living environment, are there aspects that help you with your functioning?

When you think about your surroundings, such as other people or your living environment, are there aspects that hinder you in your functioning?

Do you have certain needs related to the care of your condition? This can include specific support or treatments from healthcare providers, as well as assistance from home care for household tasks.

If yes, what kind of support from healthcare providers do you need?

If yes, which healthcare provider do you think should be involved in the care of your condition?

Can you describe to what extent you accept life with lipoedema/your functioning as it is?

Can you describe how you see yourself? (Examples: self-image)

When you think about yourself and the person you are, are there aspects that help you with your functioning?

When you think about yourself and the person you are, are there aspects that hinder you in your functioning?

What are your expectations for the future regarding your functioning?

What could you need?

Who could help you with that?

What helps you to continue functioning as you would like?

What are the things that hold you back from functioning as you would like?

What advice would you give to yourself to improve your functioning with lipoedema?

What advice would you give to others with lipoedema to improve their functioning?

What advice would you give to healthcare providers to improve the functioning of people with lipoedema?
